# Assessment of a Sensitive qPCR Assay Targeting a Multiple-Copy Gene to Detect *Orientia tsutsugamushi* DNA

**DOI:** 10.3390/tropicalmed4030113

**Published:** 2019-07-31

**Authors:** Chien-Chung Chao, Tatyana Belinskaya, Zhiwen Zhang, Le Jiang, Wei-Mei Ching

**Affiliations:** 1 Viral and Rickettsial Diseases Department, Infectious Diseases Directorate, Naval Medical Research Center, Silver Spring, MD 20910, USA; 2 Department of Preventive Medicine and Biostatistics, Uniformed Services University of the Health Sciences, Bethesda, MD 20814, USA

**Keywords:** scrub typhus, *Orientia tsutsugamushi*, qPCR, traD gene, multiple copy gene, diagnosis

## Abstract

Scrub typhus is caused by an obligated intracellular organism, *Orientia tsutsugamushi* (*Orientia*). The disease was traditionally thought to be limited in the tsutsugamushi triangle. Recently, scrub typhus has been confirmed in areas outside the triangle. Serological diagnosis of scrub typhus relies on indirect immunofluorescence assay (IFA). Molecular assays such as PCR, qPCR, loop-mediated isothermal amplification, and recombinase polymerase amplification are often targeting a single copy gene. These assays are sensitive and specific, yet they are not broadly used in clinical settings possibly due to low circulating *Orientia* in blood. In this study, we compared qPCR results using a multiple copy (traD) gene with those using a single copy (47 kDa) gene to assess the improvement of sensitivity and limit of detection. Our results demonstrate that the qPCR using the traD gene provides superior sensitivity in 15 *Orientia* strains. The limit of detection is below single *Orientia* genome equivalent and the assay retains specificity with excessive DNA from mouse, chiggers and human. The clinical utility was evaluated using confirmed scrub typhus positive and negative samples. The results show 100% sensitivity and specificity in these samples suggesting that the traD gene qPCR may be useful for clinical diagnosis of *Orientia* infection.

## 1. Introduction

Scrub typhus (ST) is an acute febrile illness caused by an obligate intracellular bacterium, *Orientia tsutsugamushi* (*Orientia*). The bacterium is transmitted to human via a bite by the larval stage (chigger) of *Orientia* harbored *Leptotrobidium* mite. The disease is prevalent in the tsutsugamushi triangle encompassing a broad Asia–Pacific region where over 1 billion people are at risk with estimated 1 million cases annually. Clinical manifestation of the disease varies from mild to severe with symptoms like eschar, fever, myalgia, maculopapular rash, lymphadenopathy, and coagulopathies that can result in circulatory system collapse [1,2]. The disease can contribute up to 20% of all acute undifferentiated febrile illnesses. Among the blood culture-negative fever patients, as high as 27% of these patients are scrub typhus positive [3,4,5]. The mortality of this high prevalence disease ranges from as low as 1.4% to as high as 70%. The outcome of the disease depends upon patients’ prior immune status, administration of proper antibiotics in a timely manner and the virulence of the *Orientia* strains [1,6,7,8]. Since the disease can be effectively treated by doxycycline, accurate and timely diagnosis can provide prompt treatment to alleviate fever and facilitate recovery of patients. The disease has recently been found outside the traditional triangle in places like Middle East, South America and Africa [9,10,11,12], highlighting the global need for a sensitive diagnostic assay.

There is no FDA cleared diagnostic assay that is commercially available. Serological assay, such as indirect immunofluorescence assay (IFA), has been the gold standard for diagnosis. However, it often requires paired sera collected weeks apart to confirm a four-fold titer increase for diagnosis. This serological assay not only suffers the high seroprevalence baseline among population in the endemic areas, it also cannot provide diagnosis in a timely fashion for prompt treatment. Therefore, various molecular assays targeting different *Orientia*-specific genes have been developed. These include qPCR/PCR assays detecting 47 kDa [13], 56 kDa [14], 16s rRNA [15], *GroEL* [16], and *OmpA* [17] genes, loop-mediated isothermal amplification (LAMP) assays detecting 47 kDa [18] and *GroEL* genes [19], and recombinase polymerase amplification (RPA) assay targeting the 47 kDa gene [20]. Keller et al. [21] have demonstrated that a multiple copy gene *traD* qPCR improves the sensitivity to detect *Orientia* DNA in a footpad infection mouse model. The utility of *traD* qPCR for clinical diagnosis has not been evaluated. Moreover, the specificity and sensitivity of the *traD* gene qPCR in various strains of *Orientia* has not been extensively studied. More recently, a multiplex qPCR assay targeting the 47 kDa and *GroEL* genes was developed and evaluated for sensitive diagnosis of scrub typhus [22]. The authors concluded that 86.5% sensitivity and 100% specificity was achieved with the additional benefit of confirming scrub typhus patients in samples with inconclusive IFA results based on a single blood sample. These results suggest a multiplex qPCR assay can serve as a more sensitive assay to diagnose scrub typhus using just a single blood sample without the need to wait for convalescent serum for IFA confirmation. 

This study aims to evaluate the performance of the *traD* qPCR assay originally developed by Keller et al. [21]. We demonstrated that the assay is broadly reactive to detect *Orientia* DNA from 15 strains previously isolated in the tsutsugamushi triangle. The detection limit was less than one *Orientia* genome equivalent. The sensitivity and specificity were not affected by the presence of chigger-mites, mouse or human genomic DNA. The assay was evaluated with a limited number of scrub typhus positive and negative samples and showed 100% sensitivity and specificity. Moreover, the assay was able to detect *Orientia* DNA in samples that were prior PCR negative but confirmed positive based on IFA. Taken together, the results demonstrate and confirm the clinical utility of *traD*-based qPCR for sensitive and specific detection of *Orientia* DNA.

## 2. Materials and Methods 

### 2.1. Sources of Orientia Genomic DNA

*Orientia* DNAs used in this study were purified from three different sources as detailed below. The first set of DNA was genomic DNA extracted from renografin-purified whole cells of 15 different strains (Table 1). The second set of genomic DNA was extracted from two chigger-mite lines (*Leptotrombidium chiangraiensis* (Lc) and *Leptotrombidum impalum* (Li)) established in Armed Forces Research Institute of Medical Sciences (AFRIMS), Bangkok. The third set of DNA was extracted from liver of mice infected by chigger-mites as described by Ching et al. [23] under the animal protocol (PN #12-12). The protocol was approved by the AFRIMS Institutional Animal Care and Use Committee and all procedures were conducted in compliance with the Animal Welfare Acts. Qiagen QIAmp DNA Mini kit (Qiagen, Germantown, MD, USA) was used for DNA extraction from whole cells suspension and mouse liver, and Invisorb Spin Tissue Mini kit from Stratec Molecular (Stratec Molecular, Berlin, Germany) was used to extract DNA from chigger-mites. Purification was performed according to the manufacturer protocols. DNA concentration was measured by NanoDrop 2000 Spectrophotometer (Thermo Fisher Scientific, Waltham, MA, USA) to ensure purity (A_260_/A_280_ > 1.8 with concentration at least > 30 ng/μL). The *Orientia* genome copy (or referred as genome equivalent, GE) of all extracted DNA was determined using qPCR targeting 47 kDa as it is a known single copy gene in *Orientia* genome. Thus one copy of the 47 kDa gene is one *Orientia* genome equivalent. This determined GE was used for sample preparation in all additional experiments when a designated GE sample was needed.

### 2.2. qPCR Condition for Single Copy 47 kDa Gene and Multiple Copy traD Gene

The qPCR primer sets for the 47 kDa (single copy) gene and the *traD* (multiple copies) gene were described before [13,21]. These primers were ordered from Eurofins MWG Operon (Louisville, KY, USA). Master mixes of QuantiFast SYBR Green PCR kit (Qiagen, Germantown, MD, USA) and RT SYBR Green Rox qPCR master mix (Qiagen, Germantown, MD, USA) were used for the multiple copy *traD* gene and the single copy 47 kDa gene qPCR, respectively. For each reaction, 0.5 μM of each primer was added in a total volume of 20 μL containing 2 μL of extracted DNA template. The qPCR cycling condition used was 95 °C for 10 min followed by 40 cycles of 95 °C for 15 s and 60 °C for 60 s in a 7500 Fast Real-Time PCR System (Applied Biosystems, Foster City, CA, USA). The plasmids for the 47 kDa and *traD* genes were prepared as described previously [20] and were used to generate copy number standard curves at a concentration range from 10^5^ to 10 copies/PCR reaction. All samples for standard curves were performed in duplicates. The qPCR efficiency for both genes varied between 90% to 105%. RNase free water was used as a negative control for both genes. 

### 2.3. Comparison of Performance of Single-Copy Gene vs. Multiple-Copy Gene Using Genomic DNA

Three different DNA sources were used to compare the results between the *traD* gene and the 47 kDa gene qPCR. The concentration of DNA extracted from different strains of *Orientia*, or two different chigger-mites, or liver collected from Lc-infected mice was determined by the 47 kDa qPCR as described previously. The concentration was adjusted to 200 copies/2 μL (i.e., 200 copies/qPCR reaction) then a serial of 1:5 dilution to achieve 200, 40, 8, 1.6 and 0.32 copies/qPCR reaction). The diluted DNA was added to qPCR reactions to quantitate the 47 kDa and *traD* genes. The copy number of both genes at different concentration was determined using known copy number of standard 47 kDa or *traD* plasmids. The cycle threshold (Ct) values of both genes at each concentration were also determined and compared. The melting curve of each amplification was evaluated to ensure consistency between each sample and standard, this was particularly the case when Ct was close to or above 35. When an inconsistent melting curve was observed for a given sample, the sample was considered as negative regardless of Ct value. The data is presented as ΔCt and was calculated by averaging the ΔCt of all dilutions or all replicates of the same dilution. For each dilution, the ΔCt was calculated by subtracting the Ct values of the *traD* gene from that of the 47 kDa gene for a given *Orientia* strain. Additionally, genomic DNA isolated from pure Karp strain of *Orientia* with known copy number of the 47 kDa gene was spiked in normal human plasma. The spiked samples were then subjected to DNA extraction. The extracted DNA was quantified by the 47 kDa gene and *traD* gene qPCR. Each DNA preparation was prepared and analyzed independently in at least five replicates. 

### 2.4. Demonstration of Specificity of traD Gene qPCR 

Several genomic DNA from related bacteria included various *Rickettsia* and *Anaplasma* in 10^5^ copy/μL was added to the qPCR reaction to determine if the *traD* gene was specific. Furthermore, the extracted DNA from Lc or Li chigger-mites, and Lc-infected mouse liver was used to demonstrate the specificity of the *traD* gene qPCR.

### 2.5. Evaluation of traD qPCR Using DNA Extracted from Confirmed Scrub Typhus Positive and Negative Patient Blood

To demonstrate the *traD* qPCR is sensitive and specific to detect *Orientia* DNA in scrub typhus confirmed patients, 2 μL of extracted DNA from scrub typhus confirmed positive patients (by IFA, PCR or qPCR), healthy individuals or patients confirmed with other infectious diseases were included in the study (PJT-15-15). 

### 2.6. Statistical Analysis

**Figure 1:**
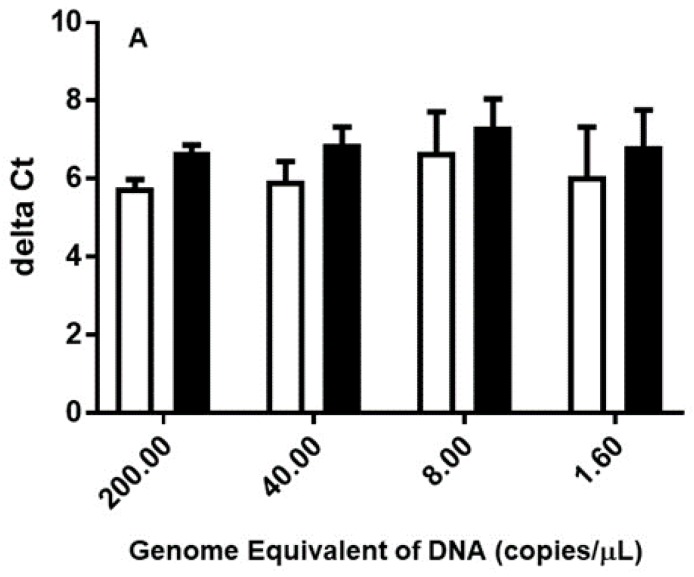

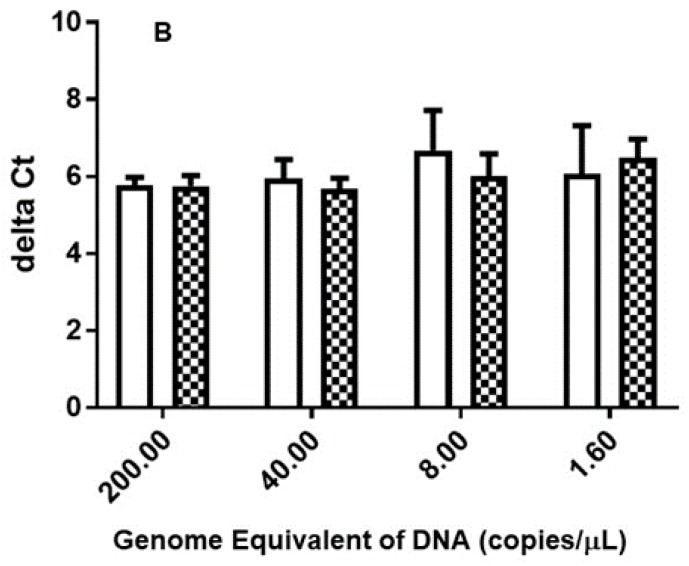
Comparison of ΔCt in *Orientia* DNA using Ct determined by the 47 kDa and *traD* qPCR. Genomic DNA was extracted from *Leptotrombidium chiangraiensis* (Lc) (open bars in Panel **A** and **B**) chigger mites, Lc chiggers infected mouse liver (black bars in Panel **A**) or *Leptotrombidum impalum* (Li) (hashed bars in Panel **B**) chigger mites as described. The extracted DNA was diluted to the appropriate concentration according to the GE determined based on the 47 kDa gene as described in Materials and Methods. ΔCt was calculated by subtracting the Ct determined using the *traD* gene from that determined using the 47 kDa gene at a given GE. The difference in ΔCt between the DNA extracted from different sources was not statistically significant.

Data was analyzed using GraphPad Prism 7.0. The statistical analysis of ΔCt displayed in Figure 1 was performed using the procedure of Benjamini, Krieger, and Yekutieli with Q = 0.01. ΔCt obtained at each DNA preparation was compared among all three different DNA sources (i.e., two different chigger-mites and chigger-infected mouse liver).

## 3. Results

### 3.1. Multiple-Copy Gene qPCR Shows Lower Detection Limit in Comparison to Single-Copy Gene in DNA Extracted from Purified Organisms, Chigger-Mites and Chigger-Mite Infected Mouse Liver

The purity of 15 strains of *Orientia* extracted from individual whole cell suspension was measured by Nanodrop spectrophotometer. Each DNA preparation (200, 40, 8, 1.6 or 0.32 copies *Orientia* GE per reaction) was analyzed for the 47 kDa and *traD* genes. The 47 kDa gene qPCR could not detect any 0.32 copies *Orientia* GE replicates and only detected some 1.6 copies *Orientia* GE replicates. The range of ΔCt (Table 1) was from as low as five to as high as ~12, suggesting the copy number of *traD* varies in different strains and it could be as low as 32 copies per GE or as high as more than 1000 copies per GE. This was similarly observed when the DNA was extracted from two different chigger-mites and from mouse liver (Figure 1A). The ΔCt obtained from chigger-mites Lc was not significantly different from that of chigger-mites Li (Figure 1B). The results in Table 2 confirm that the *traD* gene qPCR provided more consistent results when the *Orientia* genome equivalent was really low. It was clear that the *traD* gene qPCR consistently showed lower Ct in comparison to the 47 kDa gene qPCR. Additionally, at 0.4 copy/μL, only two out of seven samples showed detectable 47 kDa gene and both had Ct values close to 35. In contrast, the *traD* gene qPCR was able to detect all seven samples with very tight Ct values ranging from 30.2 to 30.5. The ΔCt among the three sets of extracted DNA was not statistically significant.

### 3.2. traD Gene qPCR Is Specific in the Presence of Excessive Chigger-Mite or Mouse Liver DNA 

The *traD* gene qPCR is specific to *Orientia* as the presence of various *Rickettsia* DNA (*R. typhi*, *R. conorii, R. rickettsii, R. belli, A. phagocytophilum*) did not give any positive results (Figure S1). In addition, the ΔCt obtained from Lc-chigger-mites infected mouse liver showed similar values as that in Lc chigger mites (Figure 1A), confirming the notion that the presence of excessive DNA from different sources (i.e., chigger-mite vs. mouse) did not affect the specificity of the traD qPCR. ΔCt was not calculated from 0.32 copies/qPCR reaction replicate as none of the 47 kDa gene qPCR gave positive results. In contrast, the *traD* qPCR results consistently showed Ct ranging from 32.75 ± 0.27 to 33.35 ± 0.93.

### 3.3. traD Gene qPCR Is Sensitive and Specific in Clinically Confirmed Scrub Typhus Positive and Negative Samples

In order to determine if the *traD* gene qPCR is clinically useful with acceptable sensitivity and specificity, DNA from 21 clinical samples were used to detect the presence of the *traD* gene. Among these clinical samples, the diagnosis was made based on combined results of IFA and PCR. The samples were diagnosed as ST positive as long as IFA or PCR results were positive. More specifically, 10 of them were confirmed scrub typhus positive by 47 kDa or 56 kDa PCR and/or IFA with the exception of scrub typhus 10. This patient was confirmed as positive due to a seroconversion even though the acute sample tested in this study was PCR and IFA negative. Six samples of confirmed other infections and five samples of healthy individuals were also included. Table 3 shows that all positive samples were *traD* gene qPCR positive and all negatives were *traD* gene qPCR negative, demonstrating 100% sensitivity and specificity (Table 4). It is worth noting that the *traD* qPCR results were consistent with the diagnosis based on the combined PCR and IFA results in spite of the fact that not all ST positive samples had consistent PCR and IFA results (e.g., scrub typhus 2, 3, 4, 5, 7, and 9). More specifically, using PCR results as the reference, the performance of *traD* qPCR was 100% sensitive and 73.3% specific due to identification of four negative PCR samples as positive (Table 4). Similarly, using IFA results as the reference, *traD* qPCR showed 100% sensitivity and 73.3% specificity (Table 4) because four samples were mis-identified as positive by *traD* qPCR. Furthermore, for patient scrub typhus 10 from which the acute sample was neither IFA nor PCR positive, this sample was *traD* qPCR positive. The fact that the *traD* gene qPCR was able to pick up four positives that were negative by prior PCR or by IFA suggest that the *traD* gene qPCR is more sensitive than either PCR or IFA to diagnose scrub typhus (Figure S2).

## 4. Discussion

The results presented a compelling evidence that the *traD* gene is specific and sensitive to detect *Orientia* in clinical samples. The specificity was tested in various DNA of closely related bacteria [21] as well as in the presence of excessive amounts of host DNA (i.e., chigger, human, and mouse). The increased sensitivity was demonstrated by its consistent detection of low GE of *Orientia* when a sensitive 47 kDa qPCR could not (Table 2). Furthermore, the increased sensitivity was corroborated by the detection of four clinically diagnosed scrub typhus cases in spite of prior negative PCR results (Table 3 and Table 4). It is believed that the multiple-copy *traD* gene contributes to this increased sensitivity. Keller et al. [21] have employed *traD* gene in their quantitation of *Orientia* in different tissues in a footpad challenge mouse model. Our results were consistent with theirs. We have previously demonstrated a slight increase of sensitivity in LAMP reaction using multiple copy genes. This was evidenced by shortening the reaction time to produce sufficient detectable fluorescent amplicons [24]. Others have shown increased sensitivity in detection of *Orientia* DNA in patient samples using a multiplex qPCR targeting two different genes [22]. All these observations confirm that the improvement of sensitivity can be achieved by multiplexing of several single copy genes or targeting a multiple copy gene. 

The specificity of the *traD* qPCR was first confirmed by performing BLAST search to determine the primers used were specific to *Orientia* [21]. The *in silico* results showed no cross-reactivity with genes of organisms that are phylogenetically close to *Orientia*. These in silico results were confirmed as no amplicons were observed when other sources of DNA were used. The presence of large amounts of other sources of DNA in the presence of low copy *Orientia* DNA did not affect specific detection of *Orientia* (Figure 1). Additionally, presence of DNA from chigger-mites, mouse, or human did not affect the observed ΔCt between the 47 kDa and traD genes. 

The inclusion of clinically diagnosed scrub typhus cases highlighted the clinical relevance of this sensitive qPCR assay. This was evident by the 100% sensitivity and specificity using a limited number of DNA extracted from well-defined clinical samples. More importantly, the *traD* qPCR was able to detect *Orientia* DNA with high confidence in those samples that were clinically diagnosed as scrub typhus in spite of negative PCR results. This finding demonstrated that the *traD* qPCR offered superior sensitivity to detect low copy number of *Orientia* that was otherwise undetectable using well accepted 47 kDa or 56 kDa gene-based PCR. This is particularly important as the circulating *Orientia* in blood is shown to be extremely low with a median of 13 copies/mL in blood in 155 patients evaluated [15]. While it is possible to extract DNA from larger volumes of blood to increase the overall load of *Orientia* for qPCR, it is not always possible in all clinical settings. It is thus conceivable that when limited volume of a blood sample is available for PCR or qPCR, insufficient amount of *Orientia* DNA extracted from the sample may lead to false negative results. In our *Orientia* infection mouse model, we have compared the tissues collected on different days post infection and found that the *traD* gene qPCR was able to detect DNA in these tissues earlier in the time course in comparison to that detection by the 47 kDa qPCR (data not shown), making this a sensitive assay in animal studies to monitor the quantity of *Orientia* as disease progresses; this is similarly observed by Keller et al. [21]. This could be an important improvement for diagnosis of scrub typhus during early phase of disease in vivo. The results in Table 3 support this notion. 

While the *traD* qPCR appears to be more sensitive with sufficient specificity, an evaluation of its clinical performance using additional confirmed scrub typhus positive and negative samples is warranted. The inclusion of patients diagnosed with known scrub typhus co-circulating diseases is also recommended. A limited number of clinical samples for evaluation was one of the limitations of current study. Nevertheless, we successfully demonstrated that the *traD* qPCR offers superior sensitivity and specificity which could significantly improve the molecular detection of *Orientia* DNA. Additionally, it is worthwhile to develop isothermal assays such as LAMP or RPA so that the assay may have its field applicability in resource-limited areas where qPCR instruments are not always available.

## 5. Conclusions 

We demonstrated that the *traD* qPCR was sensitive and specific for the detection of *Orientia* without interference from larger quantity of chigger-mites, mouse, or human genomes. The small scope evaluation using limited clinical samples clearly confirmed the clinical utility of this qPCR assay for sensitive detection of *Orientia* DNA. The increased sensitivity to detect *Orientia* DNA will improve diagnosis and consequently disease outcomes.

## Figures and Tables

**Table 1:** Variation of number of copies of the *traD* gene in various *Orientia* genomes DNA.

*Orientia* Isolate	Country of Origin	ΔCt *
AFC-27	Thailand	8.29 ± 0.72
18-030641	Malaysia	8.14 ± 0.34
18-032029	Malaysia	8.70 ± 1.24
AFC-12	Thailand	7.14 ± 1.40
Gilliam	Burma	5.09 ± 0.32
MAK 119	Taiwan	4.60 ± 0.35
MAK 243	Taiwan	9.17 ± 1.03
Karp	New Guinea	5.10 ± 0.26
AFPL12	Thailand	7.54 ± 0.49
TA763	Thailand	7.26 ± 1.19
AFC-1	Thailand	8.30 ± 0.34
Citrano	Australia	7.68 ± 1.63
Garton	Australia	11.74 ± 1.59
Boryong	South Korea	5.40 ± 0.13
Kato	Japan	7.41 ± 1.17

* The ΔCt was calculated by subtracting the cycle threshold (Ct) values of the *traD* gene from that of the 47 kDa gene for a given *Orientia* strain.

**Table 2:** Superior sensitivity of *traD* qPCR was demonstrated using purified genomic DNA spiked in normal human plasma *.

	Sample 1	Sample 2
Original DNA preparation (copy/μL in 200 μL NHS)	0.4	10
Extracted DNA (copy/μL in 50 μL water)	1.6	40
expected copy # in a qPCR reaction ^a^	3.2 (4.7, 5.9)	80 (31.3 ± 15.4)
% 47 kDa qPCR positive (range of Ct) ^b^	28.6% (33.7, 34.1)	100% (30.3–33.3)
% *traD* qPCR positive (range of Ct)	100% (30.2–30.5)	100% (25.2–26.1)
ΔCt	3.62 ^c^	5.66 ± 0.65

* The copy number of the 47 kDa gene in the purified Karp strain *Orientia* was determined by qPCR. This copy number was used to calculate the amount of purified *Orientia* DNA needed to add to the 200 μLnormal human plasma to achieve the desired concentration (i.e., 0.4 copy/μL or 10 copies/μL) for DNA extraction. Normal human plasma without spiked *Orientia* DNA was extracted simultaneously and showed no detectable signals. ^a.^ Numbers represent the expected copy number of the 47 kDa gene using 2 μL of extracted DNA in a qPCR reaction. Numbers in parentheses represent the actual copy number of the 47 kDa gene determined by qPCR from individual replicates or as mean ± standard deviation. The 47 kDa gene qPCR was considered positive if the melting curve was consistent with positive controls. ^b.^ A total of seven replicates of each concentration of *Orientia* genomic DNA were prepared and analyzed by qPCR independently. The range of Ct or individual Ct listed were for positive samples only. ^c.^ Average of two ΔCt was presented because Ct values were determined in only two of the 47 kDa qPCR experiments.

**Table 3:** Results of *traD* qPCR using clinical confirmed scrub typhus patient samples *.

Sample	Based on Melting Curve	Ct	PCR/IFA Result of Acute Sample
NHB1	Neg.	36.5	PCR(−) IFA(−)
NHB2	Neg.	Undetermined	PCR(−) IFA(−)
NHB3	Neg.	36.5	PCR(−) IFA(−)
NHB4	Neg.	38.0	PCR(−) IFA(−)
NHB5	Neg.	38.5	PCR(−) IFA(−)
Other Disease 1	Neg.	Undetermined	PCR(−) IFA(−)
Other Disease 2	Neg.	Undetermined	PCR(−) IFA(−)
Other Disease 3	Neg.	38.4	PCR(−) IFA(−)
Other Disease 4	Neg.	Undetermined	PCR(−) IFA(−)
Other Disease 5	Neg.	36.8	PCR(−) IFA(−)
Other Disease 6	Neg.	35.5	PCR(−) IFA(−)
Scrub typhus 1	Pos.	32.3	PCR(+) IFA(+)
Scrub typhus 2	Pos.	25.8	PCR(−) IFA(+)
Scrub typhus 3	Pos.	31.9	PCR(−) IFA(+)
Scrub typhus 4	Pos.	32.4	PCR(+) IFA(−)
Scrub typhus 5 ^a^	Pos.	38.3	PCR(+) IFA(−)
Scrub typhus 6	Pos.	28.1	PCR(+) IFA(+)
Scrub typhus 7	Pos.	30.2	PCR(−) IFA(+)
Scrub typhus 8	Pos.	30.3	PCR(+) IFA(+)
Scrub typhus 9	Pos.	24.7	PCR(+) IFA(−)
Scrub typhus 10 ^b^	Pos.	22.4	PCR(−) IFA(−)

*^.^ The Ct values were reported as the average of duplicate run for each sample tested. ^a.^ Scrub typhus 5 was diluted two-fold due to insufficient DNA volume to perform qPCR. ^b.^ This patient was diagnosed as scrub typhus positive by seroconversion between acute and convalescent samples. The sample used in the study is the acute sample that has negative result by PCR and IFA.

**Table 4:** Comparison of sensitivity and specificity of *traD* qPCR using IFA, PCR or results of clinical diagnosis *.

	Patients are ST Positive or Negative Based on the Results from		
	IFA	PCR	Clinical Diagnosis
Sensitivity (%)	100	100	100
Specificity (%)	73.3 ^a^	73.3 ^a^	100

* A sample was diagnosed as clinical positive if either PCR was positive, IFA was positive or a four-fold IFA titer increase between acute and convalescent samples was observed. ^a.^ The specificity was statistically significantly different from that calculated using clinical diagnosis as the determinant of ST positive or negative. (*p* < 0.01).

## Supplementary Materials

The following are available online at https://www.mdpi.com/2414-6366/4/3/113/s1: Figure S1: Amplification of *traD* gene was specific to the presence of *Orientia* DNA., Figure S2: Consistent melting curve was observed only in clinically diagnosed scrub typhus positive samples.
