# Supplementary material for: Assessment of a Sensitive qPCR Assay Targeting a Multiple-Copy Gene to Detect Orientia tsutsugamushi DNA
Source: Trop Med Infect Dis. 2019 Jul 31;4(3):113. doi: 10.3390/tropicalmed4030113 (PMC6789807; doi:10.3390/tropicalmed4030113)
Supplement: Supplementary file 1 [file tropicalmed-04-00113-s001.pdf]

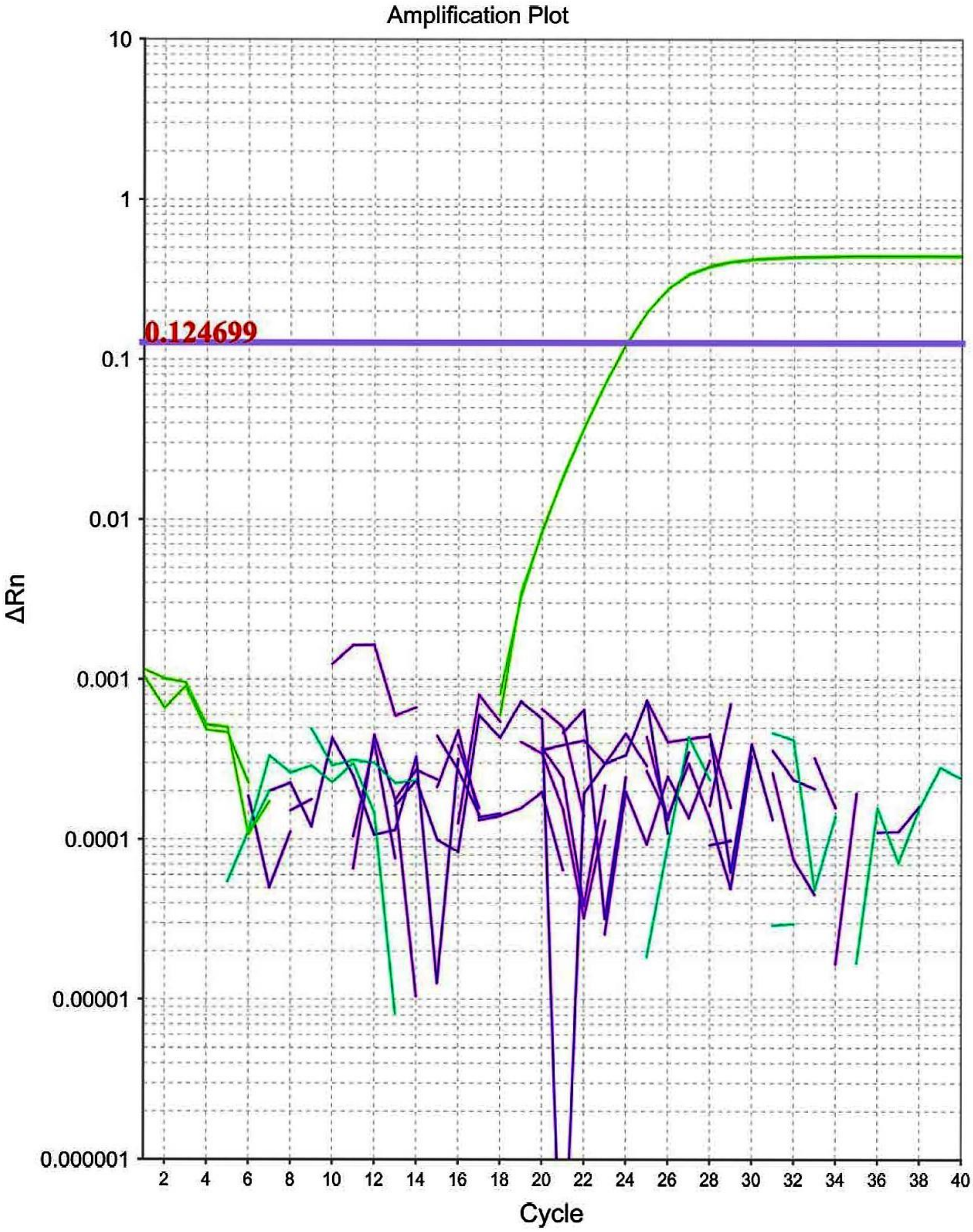

Supplemental Figure S1. Amplification of traD gene is specific to the presence of *Orientia* DNA. The traD qPCR reaction was performed in the presence of *Orientia* DNA (positive control, Kelly green line), water (negative control, Persian green line) and other closely related rickettsia DNA (purple lines) as described in **Materials and Methods**.

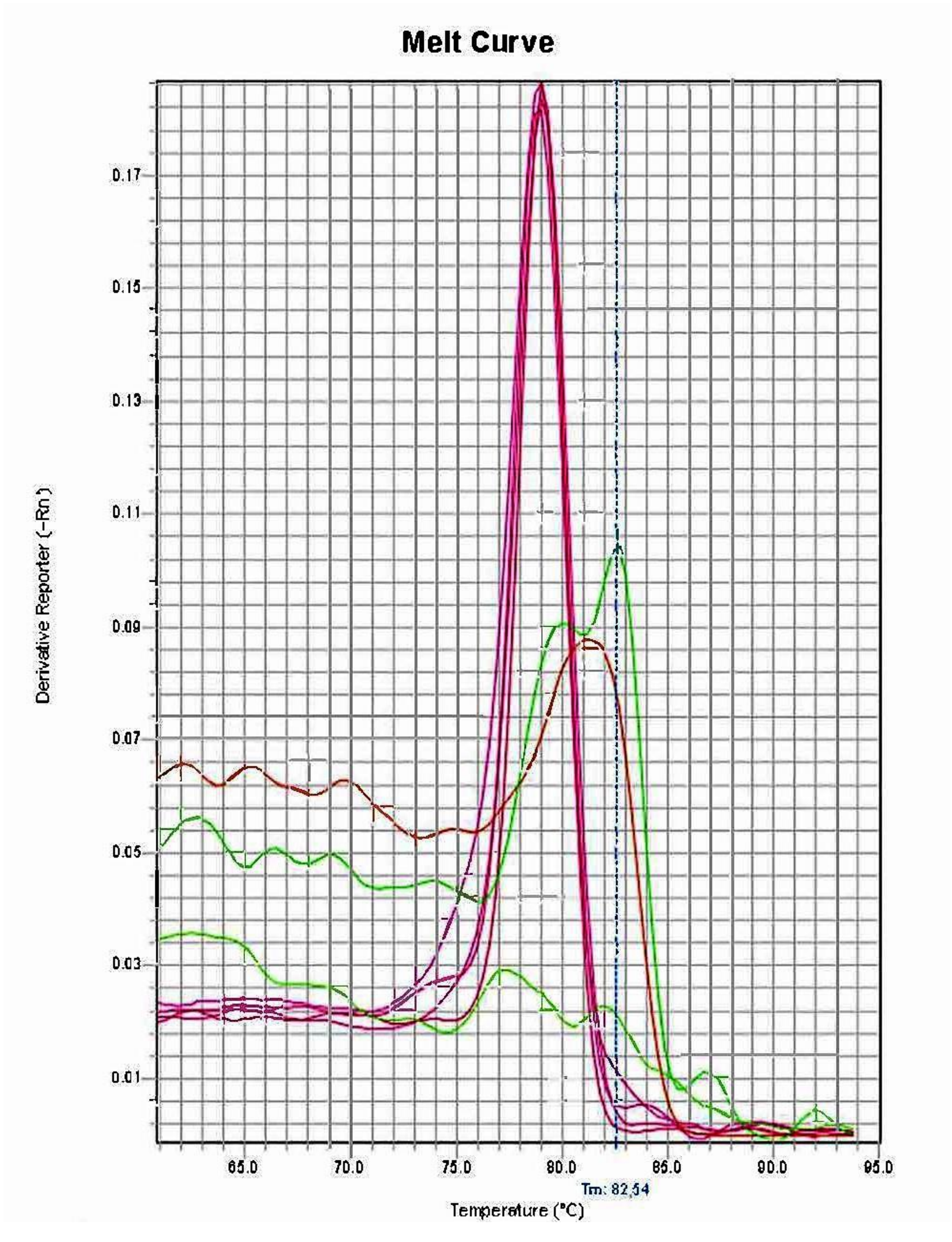

Supplemental Figure S2. Consistent melting curve is observed only in clinically diagnosed scrub typhus positive samples. The traD qPCR was performed using clinically diagnosed scrub typhus positive or negative samples as described in the Materials and Methods. Red line represents melting curve of sample NHP1, green lines represent melting curves of samples Other disease 3 and other disease 5, Ruby lines represent melting curves of samples Scrub typhus 5, 6, 8 and 10.
